# Serotonin’s Role in Mental Health Is More Complex than We Thought

**DOI:** 10.1021/acscentsci.6c00148

**Published:** 2026-02-02

**Authors:** Elizabeth Hlavinka

## Abstract

Recent research has opened the debate on whether chemical imbalances cause depression, anxiety, and other conditions.

## Graphical abstract

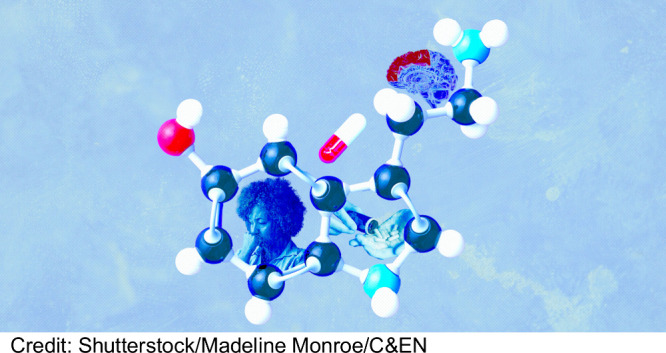

Houston considered himself lucky to have lived the majority of his life without experiencing anxiety. Still, that meant that when his panic attacks started in 2022, it felt like going from zero to 100. Sometimes the attacks would strike at work, leaving him hyperventilating and breathless. Other times they would wake him in the middle of the night, his body in a cold sweat.

“I didn’t know what a panic attack was,” says Houston, who spoke to C&EN on the condition of withholding his full name because he fears his mental health history could disqualify him from employment. “To go from nothing to full throttle was crazy.”

When Houston went to the emergency room after a panic attack, a doctor asked him whether he had been taking anything that could affect his hormones. About a week prior, Houston had stopped taking finasteride, a hormone-active anti-hair-loss cream. It appeared that what started as an attempt to curb hair loss would become a years-long struggle to get his mental health back to baseline.

**Figure:**
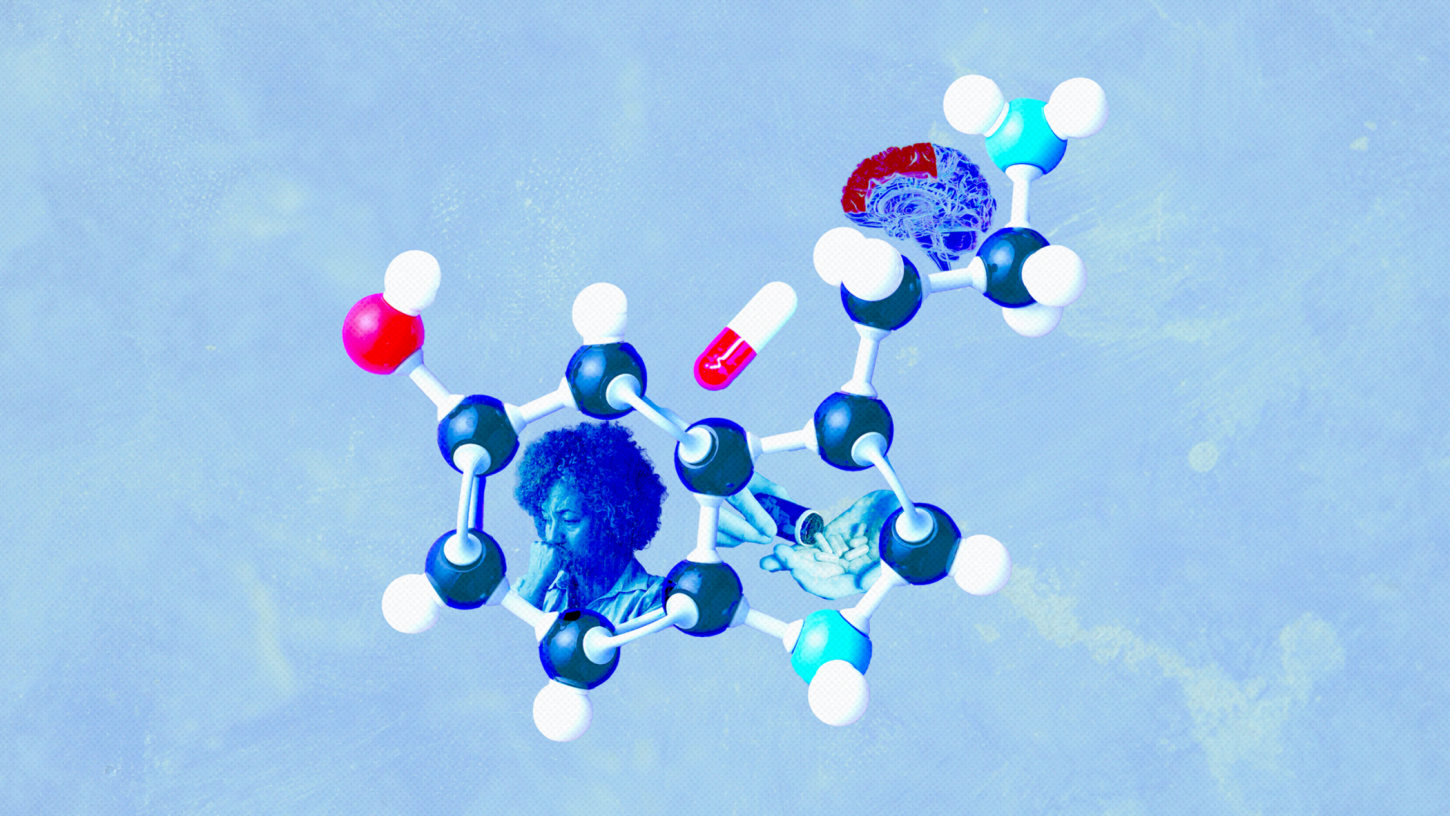
Credit: Shutterstock/Madeline Monroe/C&EN.

A significant portion of the US population—including people with drug-induced cases of panic attacks like Houston’s and some of the 2.8 million US adults with treatment-resistant depression—lives with one or more persistent mental health disorders that do not respond to drugs approved by the US Food and Drug Administration. Despite the prevalence of mental illnesses, no one really knows what causes many of them, and relatively few medications have been developed to treat conditions such as anxiety and depression.

For decades, the neurotransmitter serotonin has been thought of as one of the main culprits behind these illnesses, and selective serotonin reuptake inhibitors (SSRIs) are by far the most prescribed psychiatric medications in the US today. Yet scientists continue to debate to what extent serotonin itself is involved in these conditions—and even whether it is involved at all. New research and treatments keep revealing a more nuanced picture of how bodily systems are involved in mental illness.

The idea that conditions such as depression are caused by a chemical imbalance of serotonin in the brain has long been seen as simplistic at best. Recent studies cast doubt on the age-old serotonin hypothesis, and—guided in part by misinformation—the US federal government is putting established serotonin-based treatments under an even higher degree of scrutiny. Still, newer treatments that doctors are exploring, like psychedelics, act primarily on serotonin systems, bolstering the case for the molecule’s role in mental health conditions.

## The serotonin debate

In 2022, a controversial review in Nature added fuel to the debate over serotonin. The paper aimed to corral the data on serotonin levels and try to make sense of the findings.

The researchers analyzed studies that looked at how serotonin levels differed in people with depression and people without depression. Those studies used various measurements, including levels of serotonin metabolites in the blood and brain scans that looked at activity in the serotonin receptors and serotonin transporter protein.

Although some studies in the review did suggest links between serotonin levels and mental illness, the authors concluded there was “no consistent evidence of there being an association between serotonin and depression.”

“We’ve had this assumption that psychiatric drugs, like antidepressants, work by targeting some underlying process, like a serotonin imbalance,” says Joanna Moncrieff, one of the study’s authors and a professor of critical and social psychiatry at University College London. “But actually, this has always been an assumption.”

The review was extensively covered in the media, and many in psychiatry were quick to push back. Some argued that the review came to conclusions different from those of some of the studies that were included in it. Others said the field had always known that the serotonin hypothesis was an oversimplification and that it was never meant to fully explain mental health conditions. Many of these arguments against the paper’s conclusions were laid out in an official counterargument coauthored by 35 authors.

As the debate continued, more research exploring the link between serotonin and depression came out that contradicted the review and supported the serotonin hypothesis. In one study published soon after the 2022 paper, positron-emission tomography (PET) scans showed that people experiencing a major depressive episode released less serotonin than volunteers without depression in a control group when both groups were given a drug that normally boosts serotonin.

Although the PET study involved only 17 patients—and many of them also had Parkinson’s disease, which could affect the results—this imaging technique could help scientists more accurately observe how serotonin fluctuates in the brain in conditions such as depression.

Whether or not serotonin is the primary player in mental illnesses, it’s worth noting that SSRIs have helped many people get their lives back after struggling with conditions like depression or panic disorders that make it difficult for them to work, socialize, or seek further help. SSRIs are also more tolerable and carry a lower risk for fatal overdose compared with older treatments, such as monoamine oxidase inhibitors (MAOIs) and tricyclic antidepressants.

Still, they don’t work for everyone. Researchers estimate that at least 30% of people with depression will not respond to various types of SSRIs, making it clear that things outside of the serotonin system are at play too.

“Serotonin is probably playing a role in depression, but it’s not the main factor,” says Philip Cowen, a professor of psychopharmacology at the University of Oxford and one of the coauthors of the counterargument.

Now the debate over SSRIs has entered the mainstream—and with it large amounts of disinformation. In February 2025, the Donald J. Trump administration went so far as to initiate an investigation of SSRIs and other psychiatric treatments, insinuating that these medications are dangerous.

This investigation, however, does not seem to be looking into the role that serotonin plays in depression. Instead, Secretary of Health and Human Services, Robert F. Kennedy Jr., has raised inaccurate narratives related to SSRIs, exaggerating how addictive antidepressants are and falsely linking them to violent behavior.

## The birth of the chemical imbalance idea

In 1967, English psychiatrist Alec Coppen proposed the serotonin theory of depression, which suggested that a lack of serotonin in the brain was one cause of the disorder. This idea was in part based on a study he and two colleagues had conducted 4 years prior.

That study found that depressive symptoms were reduced in patients taking monoamine oxidase inhibitors (MAOIs), the most common antidepressants of the time, supplemented with serotonin’s precursor, tryptophan. Coppen hypothesized that adding tryptophan increased serotonin levels, which in turn reduced depression.

If that were true, it would make sense that reducing tryptophan would induce depression. Yet in some of Cowen’s research in 2005, there was no change in depressive symptoms in people who had depleted tryptophan levels, though study participants with a history of depression did experience more severe changes in mood.

“If you have other risk factors for depression, and if you’ve been depressed, then serotonin may be of consequence,” Cowen says. “But by itself, [a low serotonin level is] not enough to cause depression.”

Despite the complexities and nuances of the serotonin system, the simplistic idea that depression was caused by serotonin deficiency became central to marketing materials for SSRIs.

Compared with MAOI drugs, which boost levels of several neurotransmitters, including serotonin, norepinephrine, and dopamine, SSRIs are more “selective” in targeting only the serotonin system. They block the receptors that let serotonin into nerve cells. That causes the serotonin to linger outside the cells and stimulate the serotonin receptors for a longer period of time.

**Figure:**
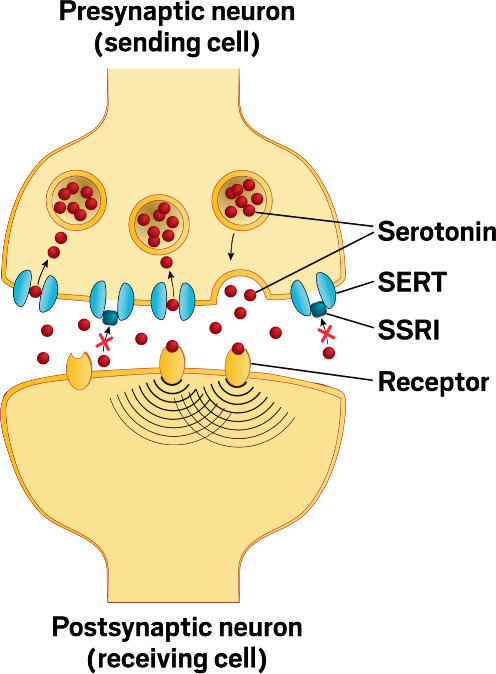
**How SSRIs supposedly work.** Selective serotonin reuptake inhibitors (SSRIs) are thought to work by blocking the serotonin transporter (SERT), a protein that sucks leftover serotonin back into the neuron from the synapse. This supposedly leads to higher serotonin levels in the synapse and affects depression. Credit: Yang H. Ku/C&EN/Shutterstock.

This mechanism is often interpreted to mean that SSRIs increase serotonin levels, but the reality is far more complex. For example, some studies have shown that serotonin levels in plasma decrease after SSRI treatment.

One of the main points of contention in the serotonin debate is that measuring the activity of neurotransmitters directly in the brain has historically been challenging. As a result, many studies rely on indirect measurements of serotonin, like the presence of tryptophan. But the brain is like an interconnected web, and adding or removing one neurochemical sets off a chain of events that makes it difficult to attribute any one outcome to a singular system.

“It’s really a myth to say SSRIs work by increasing brain serotonin because which compartment are you talking about and who’s measured it? Nobody,” says Anne Andrews, a professor of psychiatry, chemistry, and biochemistry at the University of California, Los Angeles. “We don’t really know throughout these complex adaptive processes what’s actually happening.”

## Many potential pathways to depression

Cases like Houston’s make it clear that there are more pathways to mental illness than previously thought. The finasteride Houston was using was not intended to have an effect on his serotonin levels, and it’s unclear whether it did. The anti-hair-loss drug is meant to block a pathway that converts dihydrotestosterone to testosterone.

When Houston stopped using the cream, he was thrown into postfinasteride syndrome, which is a set of persistent psychiatric symptoms officially recognized by the National Institutes of Health in 2015. Because this is a drug-induced condition, Houston’s psychiatrist did not recommend SSRIs for treatment, he says.

As scientists start to explore alternative treatments and neurochemical pathways that could be influencing depression and other mental illnesses, they are finding links to the brain’s neuroplasticity, or the ability for the brain to form and reorganize new synaptic connections.

For example, long-term SSRI use is thought to work primarily on serotonin, but it has also been associated with increased activity of a protein that promotes neural plasticity called brain-derived neurotrophic factor (BDNF). Other psychedelic antidepressant treatments such as ketamine and psilocybin also increase BDNF activity.

This makes sense when put together with the clinical presentation of depression, in which patients get stuck in oppressive thought patterns. While this is just another hypothesis for how antidepressants work, the idea is that increasing neuroplasticity can help the mind escape those thought patterns.

In line with this thinking, one alternative hypothesis is that the antidepressant effect of SSRIs comes not from the inhibition of serotonin reuptake but rather how our brain adapts to the drugs.

And even if neuroplasticity and BDNF activity were the primary drivers of mental illness, that still wouldn’t rule out serotonin’s importance. Studies in rodents have shown that the formation, function, and survival of serotonin in the brain rely on BDNF. Other mental health treatments point back to serotonin too. It has long been known that psilocybin directly targets a serotonin receptor, while ketamine increases activity of the glutamate system, which boosts extracellular serotonin.

**Figure:**
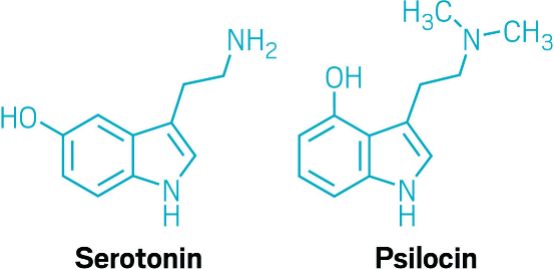
Serotonin and psilocin bind to the same receptor and bear structural similarities. Psilocybin, an antidepression drug candidate, turns into psilocin in the body.

Separating serotonin from other bodily systems is nearly impossible because its role is precisely to connect disparate bodily systems. Serotonin relays messages from the brain to the body, telling it how to react to stimuli. It plays a role in detecting and responding to threats, and it also helps regulate mood, platelet function in the blood, and digestion, for example.

Adding to the complexity, our cells contain 15 known types of serotonin receptors, which is twice as many as most of the brain’s other neuromodulator systems. Yet just 2% of the body’s serotonin is located in the brain; 8% is in platelets, and the remaining 90% is in the gut.

## Gut serotonin and mental illness

Often called the “second brain,” the digestive tract is profoundly complex, with at least as many neurons as the spinal cord.

Many mood disorders are accompanied by gut-related symptoms. In Houston’s case, his panic attacks were accompanied by diarrhea, acid reflux, and a loss of appetite.

Theories on how gut bacteria affect mood disorders—and vice versa—posit that an imbalance of neurotransmitters and hormones in the gut could lead to mental health symptoms. The psychiatric field is blooming with questions about whether gut bacteria can influence the immune system, and some recent research suggests the bacteria may be producing substances like short-chain fatty acids that can influence brain activity.

The exploration of how microbiota affect mental illness was spurred by a study called “Transferring the blues,” in which a research team at University College Cork transplanted microbiota from people with depression into rodents and observed behavioral changes linked to depression. This led to the question of whether the reverse was possible: Could transplanting microbiota from healthy patients into those with depression improve symptoms?

In 2023, Houston was desperate. Changes to his diet had reduced the number of panic attacks he experienced from multiple per day to about one per week, but the attacks hadn’t subsided completely. About 1 year after his first panic attacks, he decided to try a therapy called a fecal microbiota transplant (FMT).

FMTs involve consuming capsules containing stool from a healthy donor or transplanting the fecal microbiota manually through a colonoscopy or nasal tube. The therapy has been used to treat digestive issues, but some case reports have also shown reduced symptoms of depression and bipolar disorder. Clinical trials are underway to test their effectiveness in treating depression and obsessive-compulsive disorder, but large-scale studies are lacking. “I get that it’s not FDA-approved, but it was an FDA-approved drug that did this to me,” Houston says.

Houston had an immune response to the treatment, experiencing a fever and chills for the first few days and diarrhea for about a week after. About 1 month after Houston underwent the FMT, his panic attacks went away.

Because the microbiome is so vast, researchers are looking for more-specific targets in the gut that could help improve mood. In a 2025 study, researchers found that increasing serotonin levels in the gut epithelium in rodents was associated with reduced symptoms of anxiety and depression.

“By targeting the gut epithelium, you may be able to treat anxiety and depression without directly impacting the enteric nervous system or the brain, which may have off-target effects,” says study author Kara Gross Margolis, director of the New York University Pain Research Center and a pediatric gastroenterologist at New York University-Langone Medical Center.

Once again, scientists have discovered a potentially promising therapy that likely involves serotonin.

If the strength of a hypothesis is measured by the amount of research it spurs, one could argue that Coppen’s idea was a great one.

“I’ve been trying to get away from working on serotonin for a long time,” says John F. Cryan, author of the 2016 microbiota transplant study. “And I can’t because every time I leave serotonin, it keeps coming back.”

*Elizabeth Hlavinka is a freelance contributor to* Chemical & Engineering News *, an independent news publication of the American Chemical Society*.

